# Response to Comments on the CAG/CCC Position Statement on Biosimilars for Inflammatory Bowel Disease

**DOI:** 10.1093/jcag/gwaa004

**Published:** 2020-04-18

**Authors:** Paul Moayyedi, Eric I Benchimol, David Armstrong, Cathy Yuan, Aida Fernandes, Grigorios I Leontiadis

**Affiliations:** 1 Division of Gastroenterology and Farncombe Family Digestive Health Research Institute, McMaster University, Hamilton, Ontario, Canada; 2 Children’s Hospital of Eastern Ontario (CHEO) Inflammatory Bowel Disease Centre, Division of Gastroenterology, Hepatology and Nutrition and CHEO Research Institute, Ottawa, Ontario, Canada; 3 Department of Pediatrics and School of Epidemiology and Public Health, University of Ottawa, Ottawa, Ontario, Canada

We thank Drs. Bassett and Musini for their interest in the joint Canadian Association of Gastroenterology (CAG) and Crohn’s and Colitis Canada (CCC) Position Statement on biosimilars for the treatment of inflammatory bowel disease (IBD) (1). They raise four concerns, claiming that our paper contains ‘serious flaws’ and that this ‘negates the paper’s assertions’ that switching from the originator to the biosimilar is not recommended in the majority of patients. We disagree with their concerns and welcome the opportunity to expand on the points they raise.

They raise the issue of including abstracts in our systematic review of the literature on the grounds that these cannot be independently evaluated and verified. Empirical data show that there are only minor differences between an abstract and the full paper (2); so, their concerns are not supported by available data. Furthermore, in the setting of limited randomized controlled trial (RCT) evidence, it is important to include conference abstracts so that all available evidence can be evaluated (3). However, in our analysis, the debate on whether conference abstracts should be included is irrelevant. Figures 1 and 2 show clearly that exclusion of the abstract in a sensitivity analysis would not change the results or the certainty of evidence.

Drs. Bassett and Musini suggest that we should not have synthesized the data relating to switching from originator to biosimilar as the studies reported different endpoints. This is a common debate with respect to meta-analyses as it is rare that trials use exactly the same methodology or report the same endpoints. It is always a judgement call as to whether to synthesize such data. Both trials evaluated disease worsening, but one also included withdrawal rates, which is the ultimate assessment of whether a new therapy is needed and often relates to disease worsening. We felt it was appropriate to combine these data as they pertain to a similar outcome, but we did downrate the certainty of evidence for this reason (Table 3). However, again, this is a debate that is irrelevant, as a sensitivity analysis, in which each trial is assessed separately, does not change the conclusions. The two RCTs independently suggest worse outcomes after a biosimilar switch so, regardless of whether one ‘lumps or splits’ the results, the conclusion remains the same as does the certainty of evidence (very low).

The authors criticized us for omitting an RCT (4) that showed no differences in rates of clinical remission, loss of response or disease worsening. We included Ye et al. (4) in the assessment of biosimilar versus originator treatment in anti-TNF naive IBD patients. We did not include this trial in the meta-analysis on switching because it did not fulfil two important inclusion criteria; first, the trial did not recruit ‘IBD patients in remission while on originator anti-TNF’ because 39% of the patients were not in clinical remission when they were switched, at week 30 and, second, the 24-week follow-up period was too short to detect a difference in outcomes, unlike the two included RCTs which reported 1-year outcomes.

Drs. Bassett and Musini were concerned that we had not included noncontrolled studies as many other systematic reviews had done. While the quality of evidence from controlled studies may be low, due to flaws in study design or analysis, evidence from cohort studies is inherently of lower quality, particularly if there is no comparator group; we, therefore, made an a priori decision to include only RCTs and cohort studies with a comparator and this is the main methodological strength of our approach. Our goal was to evaluate the best-available evidence objectively, according to current best practice as a basis for our recommendation rather than to seek evidence that would support a predefined position. This notwithstanding, we did identify and evaluate the surprisingly large number of systematic reviews that have already assessed this topic, and all but one (5) failed to separate the majority of observational studies that did not have a control group from the small number that did. All “evidence” is not created equally as Drs. Bassett and Musini imply in their critique of the controlled studies that we selected for analysis; their concern should be equally valid when considering the inclusion of uncontrolled data. No inferences can be made on the safety of biosimilars without an originator comparator group unless the differences are extremely large. It may be appropriate to downgrade the strength of evidence from RCTs but not, then, to trump this with even lower quality evidence from uncontrolled studies.

A weak recommendation against switching suggests that the majority of patients should not undertake this approach, but we must emphasize that it may be appropriate, in a minority of instances after careful discussion with the patient. This is a patient-oriented approach which has the added advantage of providing more information regarding the efficacy and safety of the biosimilar in patients who are switched from the originator product. Simply declaring that such a switch is ‘safe’ without any regard to evidence-based medicine is not in the interest of patients and, furthermore, it may prove to be less cost-effective than third-party payers had hoped. We can be more certain of the outcome if we have more evidence and this can only be achieved if such a switch is done in a more graduated way with careful pharmacovigilance.

Finally, we would like to acknowledge a potential conflict of interest for the authors of the letter, as declared in the third reference of their communication: “The Therapeutics Initiative is funded by the BC Ministry of Health through a grant to the University of BC”. This suggests that they may be biased in favour of a BC Ministry perspective. All but one of the authors of our paper have no pharmaceutical company conflicts of interest but most of us treat patients with IBD and we therefore may be biased in favour of patient care with less focus on the cost of that care. Both perspectives are clearly important, and we suggest that future policy decisions be made with strong, transparent representation from IBD patients and clinicians so that all relevant voices can be heard, and decisions made that reflect all points of view.
